# Combining Geostatistics with Moran’s I Analysis for Mapping Soil Heavy Metals in Beijing, China

**DOI:** 10.3390/ijerph9030995

**Published:** 2012-03-19

**Authors:** Xiao-Ni Huo, Hong Li, Dan-Feng Sun, Lian-Di Zhou, Bao-Guo Li

**Affiliations:** 1 Beijing Academy of Agriculture and Forestry, Beijing 100089, China; Email: Liandizhou@126.com; 2 Department of Environmental Engineering, Taiyuan University, Taiyuan 030009, China; Email: hxnsky@126.com; 3 Department of Land Resources and Management, College of Natural Resources and Environment Science, China Agricultural University, Beijing 100193, China; Email: sundf@cau.edu.cn (D.-F.S.); libg@cau.edu.cn (B.-G.L.)

**Keywords:** geostatistics, Moran’s I analysis, heavy metals, Beijing

## Abstract

Production of high quality interpolation maps of heavy metals is important for risk assessment of environmental pollution. In this paper, the spatial correlation characteristics information obtained from Moran’s I analysis was used to supplement the traditional geostatistics. According to Moran’s I analysis, four characteristics distances were obtained and used as the active lag distance to calculate the semivariance. Validation of the optimality of semivariance demonstrated that using the two distances where the Moran’s I and the standardized Moran’s I, *Z(I)* reached a maximum as the active lag distance can improve the fitting accuracy of semivariance. Then, spatial interpolation was produced based on the two distances and their nested model. The comparative analysis of estimation accuracy and the measured and predicted pollution status showed that the method combining geostatistics with Moran’s I analysis was better than traditional geostatistics. Thus, Moran’s I analysis is a useful complement for geostatistics to improve the spatial interpolation accuracy of heavy metals.

## 1. Introduction

Heavy metal pollution in agricultural soils is becoming an urgent problem worldwide, due to increasing intensive anthropogenic activities, such as the discharge of wastes from metal processing plants, burning of fossil fuels and pesticide use. Excessive accumulation of heavy metals in agricultural soils can also be a source of pollution of surface and ground waters, living organisms, sediments, and oceans. Thus, mapping the spatial distribution of heavy metals in soils is critical for risk assessment of potential environmental pollution and for establishing protocols for pollution remediation, in particular, for China, with the recent three decades of intense economic development, the soil heavy metals’ pollution is now in a high risk state.

Geostatistics, providing a technique of semivariance to quantify the spatial patterns of soil parameters, is being increasingly adopted for spatial pattern analysis of heavy metals [1,2]. One criterion for evaluating the spatial dependence of spatial variables is imparted by the nugget/sill ratio [3,4], without the significance test of spatial dependence. In geostatistics, the active lag distance specifies the range over which semivariance can be calculated, which is usually about half of the maximum separation distance. However, this is only an empirical method [5]. In practice, due to the complexity of spatial data, it is difficult to perform iterative trial runs to determine a suitable active lag distance in order to generate a relatively stable and better-fitting theoretical semivariance without *a priori* knowledge [6]. In addition, spatial outliers in a dataset often make the semivariogram exhibit erratic behavior [7]. As a result, outliers are often deleted from spatial predictions [6,7]. However, in soil heavy metal evaluations, it is dangerous to ignore outliers, as these may actually represent potentially severely pollution areas [8].

Spatial autocorrelation analysis is another alternative method that has been widely used to explore the spatial pattern of variables in many fields [9,10,11]. Moran’s I is used to estimate the strength of the spatial correlation, and the significance of the spatial correlation can be tested [7,11]. Furthermore, spatial autocorrelation analysis can identify spatial clusters (positive autocorrelation) and spatial outliers (negative autocorrelation) of a regionalized variable [12]. Consequently, Huo *et al.* adopted Moran’s I to describe the spatial distribution pattern of heavy metals in Beijing cultivated soils [8].

Although spatial autocorrelation analysis cannot be used for estimation of unsampled areas, we recognized it could provide useful information for spatial variable mapping, if combined with a kriging method, for the production of high quality distribution maps. Therefore, taking heavy metals in Beijing agricultural soils as a case study, the primary objectives of this research were: (1) to compare spatial autocorrelation analysis and geostatistics for identifying the spatial pattern of heavy metals; and (2) to use the Moran’s I analysis results as the *a priori* knowledge for geostatistical interpolation, and to evaluate their effects on the estimation accuracy of heavy metal kriging mapping, focusing particularly on their influence on pollution status estimations.

## 2. Materials and Methods

### 2.1. Soil Sampling and Analysis

**Figure 1:**
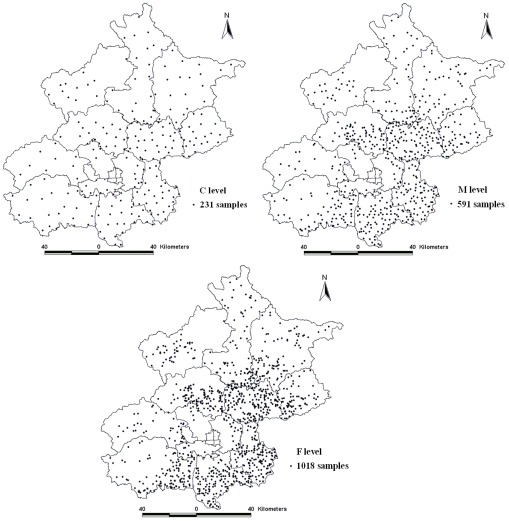
The distribution of soil samples at three levels.

To investigate the pollution status of heavy metals in Beijing agricultural areas, a large-scale soil sampling project was conducted after the crop harvest in the autumn of 2006. According to the agricultural land distribution and land use type maps of Beijing, a non-uniform distribution of the stratified sampling technique was adopted to collect samples and ensure the representativeness of samples. The sampling strategy was divided into three steps to collect a total of 1,018 samples. First, 231 soil samples were collected from the entire study area, with uniform sampling being the low sampling density (C). Secondly, another 360 soil samples were added from areas with more agricultural soils to create the medium sampling density (M). Third, 427 soil samples were further collected on the basis of the two previous samplings and the agricultural soils to make a high sampling density (F). Figure 1 shows the distribution of soil samples at the three sampling densities.

For each sample, five surface soil (0~20 cm) sites were sampled within 10 × 10 m square areas and then mixed. A Global Positioning System was used to precisely locate each sampling position (latitude and longitude); and a total of 1 kg of mixed soil per sample was collected. All soil samples were collected using a stainless steel spade and a scoop made from bamboo and then stored in polyethylene bags. The soil samples were air-dried, crushed in an agate mortar, and then passed through a 100-mesh nylon sieve. The concentrations of eight heavy metals, including Cr, Ni, Cu, Zn, As, Cd, Pb, and Hg, were analyzed in the soil samples following the Chinese Environmental Quality Standard for Soils (GB15618-1995). After digesting the samples with a mixture of HCl, HNO_3_ and HClO_4_, the Cr, Ni, Cu, and Zn concentrations were analyzed by flame atomic absorption spectrophotometry, Pb and Cd were analyzed by graphite furnace atomic absorption spectrophotometry, and the As concentration was determined by potassium borohydride-silver nitrate spectrophotometry. In addition, the Hg concentration was analyzed by cold atomic absorption spectrophotometry after the samples were digested with a mixture of H_2_SO_4_, HNO_3_ and KMnO_4_. During processing, all samples were handled carefully to avoid input or loss of trace elements during preparation and analysis.

### 2.2. Spatial Autocorrelation

Spatial autocorrelation is an assessment of the correlation of a variable in reference to spatial location of the variable, which is a match between location similarity and attribute similarity [13]. Moran’s I is the more popular test statistic for spatial autocorrelation. Global Moran’s I examines whether spatial correlation exists or not over an entire region, and it is calculated as follow as [14]:


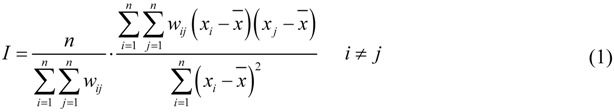


where 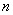 is the number of observations of the whole region, 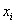 and 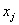 are the observations at locations of 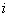 and 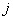, 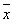 is the mean of 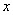, and 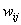, an element of spatial weights matrix 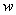, is the spatial weight between locations of 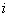 and 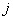. The value of Moran’s I generally varies between 1 and −1. Positive autocorrelation in the data translates into positive values of Moran’s I; negative autocorrelation produces negative values. No autocorrelation results in a value close to 0 [11].

The selection of neighbors is formally specified in the weights matrix 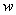, which depicts the relationship between an element and its surrounding elements. A distance-based weight matrix was adopted in this study, and each distance class is specified as a threshold distance, such that all locations within the given distance are considered to be “neighbors” (the value not equal to zero in the matrix) in the distance-based weight matrix. Usually, normal approximation as a precondition, global Moran’s I can be standardized to 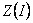, and 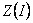 is calculated as [14,15]:


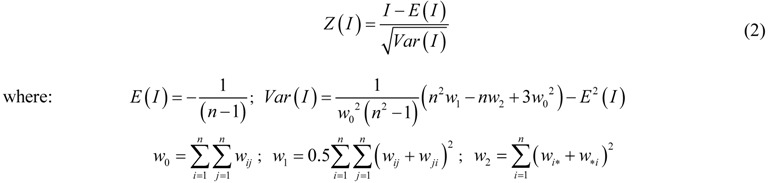


where 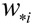 is the sum of all weights located in the row 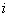, 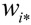 is the sum of all weights in the column 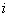. The threshold of 1.96 was applied to test the significance level of 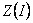. If 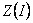 was greater than 1.96 or smaller than −1.96, it implied that the spatial autocorrelation was significant [7].

The spatial correlogram is a graph where the Moran’s I is plotted in ordinate, against distances among localities (in abscissa). According to Legendre and Fortin, the spatial correlogram can be standardized into a standardized correlogram, in which the ordinate is the standardized Moran’s I, *Z(I)* [16]. The shape of the standardized correlogram provides indications about the spatial pattern (spatial clusters and spatial outliers) and spatial correlation distance of a variable [9,16]. However, the standardized correlogram often has one or more positive correlation ranges. Zhang *et al.* explained that the closer positive correlation range represents the average size of the zone of spatial clusters, that is, the spatial correlation distance [17].

Local Moran’s I is a local test statistic for spatial autocorrelation, which is used to identify the locations of spatial clusters and spatial outliers. It is computed as follows:


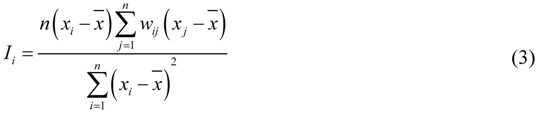


The notations in Equation (3) are as described for Equation (1), but the corresponding values are from the local neighboring region. For more details of Moran’s I principles and methods, see the references [12,14]. With the local Moran’s I statistic analysis, five categories of local spatial autocorrelation can be distinguished. Two of these are spatial clusters, including high values surrounded by high values (High-high), and low values surrounded by low values (Low-low) types. Two are spatial outliers, including high values surrounded by low values (High-low) and low values surrounded by high values (Low-high). The last type is the spatial randomness that is without significant spatial patterns at corresponding weight matrix. 

### 2.3. Geostatistics Method

Geostatistics uses the technique of semivariance to measure the spatial variability of a regionalized variable, and provides input parameters for the spatial interpolation of kriging [18]. It relates the semivariance, *γ(h)*, which is computed as half the average squared difference between the components of data pairs [19]:


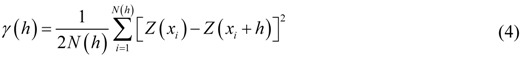


where 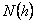 is the number of data pairs separated by a distance 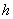, and 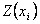 is the measured value of the variable 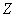 at location of 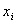, and 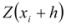 is the measured value of the variable *Z* at location 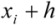. For irregular sampling, it is rare for the distance between the sample pairs to be exactly equal to *h*, therefore, the lag distance *h* is often represented by a distance band.

A variogram plot can be acquired by calculating variogram at different lags. Data pairs were grouped into lag “bins” and Equation (4) was used to calculate the variogram for that bin. The mean lag of all the pairs in a particular bin was used as the representative lag for that bin. 

The variogram plot is fitted with a theoretical model, such as spherical, exponential, Gaussian, linear and power models. In this study, the exponential model was selected.

The exponential function is:


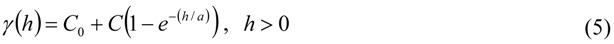


where 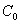 is the nugget variance (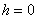), represents the experimental error and field variation within the minimum sampling spacing. Typical, the semivariance increase with increasing lag distances to approach or attain a maximum value or still (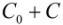) equivalent to the population variance. *C* is the structural variance and *a* is the spatial range across which the data exhibit spatial correlation. 

Due to the complexity of spatial data, its spatial variability usually needs be described using two or more theoretical semivariances. This is the so-called nested model, which is described by the following equation [20]:


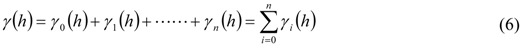


where 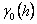 is the nugget value of the nested model, which is usually considered to be the spatial variability that cannot be described at the smallest sampling scale, and 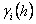 is the semivariance at different scales.

The fitted model provides information about the spatial structure as well as input parameters for kriging interpolation. Kriging is a linear interpolation technique that provides a linear unbiased estimate for spatial variables, which can be depicted as follows:


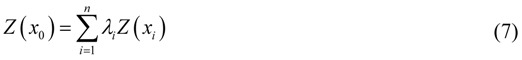


where 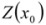 is the predicted value at location of 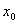, 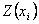 is the measured value at location of 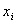, 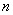 is the number of sites within the search neighborhood used for the estimation. Contrary to other methods (such as inverse distance weighted), the weighting function 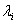 is no longer arbitrary; rather, it is calculated based on the parameters of the semivariogram model. To ensure that the estimate is unbiased, the weights need to sum to one:


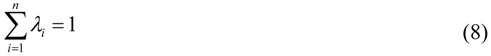


and the estimation errors (or kriging variances) need to be minimized.

With wide and increasing applications of spatial interpolation methods, there is a growing concern about their accuracy and precision. Accuracy of spatial interpolation was evaluated through cross-validation approach. Commonly used error measures include: mean error (ME), mean absolute error (MAE), mean squared error (MSE) and root mean squared error (RMSE). Willmott suggests that MAE and RMSE are among the “best” overall measures of model performance [21]. RMSE provides a measure of error size, but it is sensitive to outliers as it places a lot of weight on large errors [22]. MAE provides an absolute measure of the size of the error, and it is less sensitive to extreme values [23]. 

MAE is calculated as:


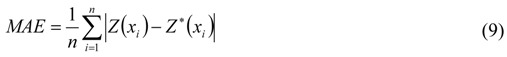


RMSE can be calculated as:


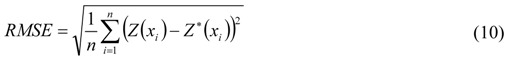


Because MAE does not reveal the magnitude of error that might occur at any point, MSE will be calculated [24] as:


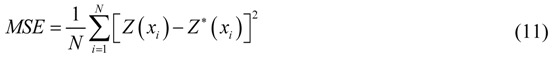


where 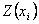, 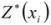 is the observed and predicted value at location of 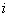, respectively. Smaller MAE values indicate few errors. The RMSE can be used to compare different methods by seeing how closely predicted values match the observed values, the smaller the RMSE the better. Squaring the difference at any point gives an indication of the magnitude. Smaller MSE values indicate more accurate point-by-point estimation.

### 2.4. Data Treatment with Computer Software

Soil samples were stored using the ArcView 3.2 software to create a spatial database. The spatial autocorrelation analysis was conducted using Geoda095i software. The experimental semivariance models were constructed using GS+5.3, while kriging was performed using the geostatistical analyst extension of ArcGIS 8.3.

As in conventional statistics, a normal distribution for the variable under study is desirable in linear geostatistics. Even though normality may not be strictly required, serious violation of normality, such as too high skewness and outliers, can impair the variorum structure and the kriging results. It is often observed that environmental variables are lognormal [1], and data transformation is necessary to normalize such data sets.

The normality tests of the eight heavy metals for the 1,018 samples were performed as described by Huo *et al.* [25]. Compared with the raw data sets, the log-transformation significantly reduced the skewness values of data sets towards “0”. However, because of data sets with many duplicate values or multi-modals, Cu, As, Cd, and Pb still cannot pass the Kolmogorov-Smirnov test for normality (K-S p) after log-transformation. As a result, further analysis focused on Cr, Ni, Zn, and Hg. In order to compare with geostatistics, the data after log-transformation were used in the Moran’s I analysis.

## 3. Results and Discussion

### 3.1. Spatial Pattern Analysis of Heavy Metals Using the Spatial Autocorrelation Analysis

**Figure 2:**
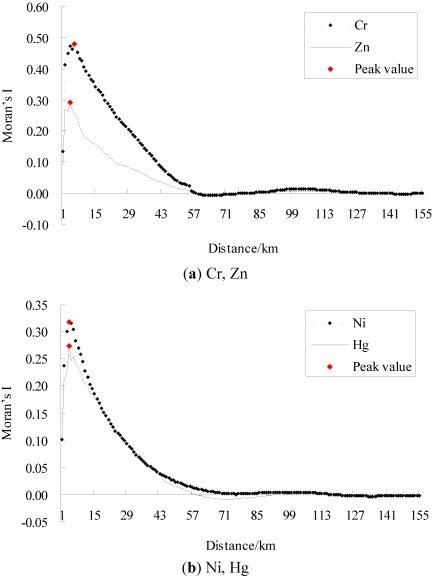
Raw spatial correlograms of heavy metals (**a**) Cr, Zn, (**b**) Ni, Hg.

**Figure 3:**
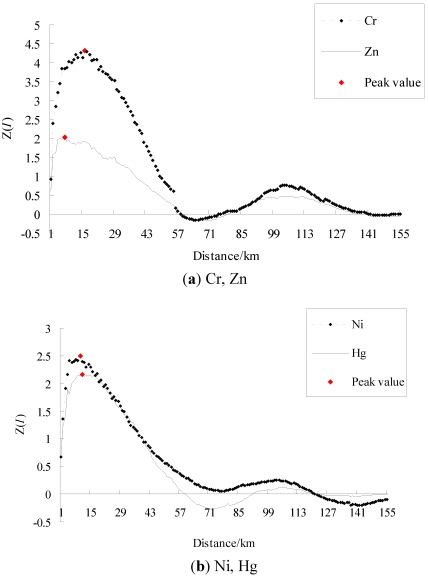
Standardized spatial correlograms of heavy metals (**a**) Cr, Zn, (**b**) Ni, Hg.

In general, the higher the absolute value of Moran’s I is, the stronger a spatial autocorrelation exists, and the larger the absolute value of standardized Moran’s I is, the more significant a spatial structure exists. Figure 2 and Figure 3 represent the raw and standardized spatial correlograms of Cr, Ni, Zn, and Hg, respectively. Table 1 and Table 2 show the spatial autocorrelation characteristics of the four heavy metals, and the critical distance of weight matrix is the distance where the Moran’s I and the standardized Moran’s I, *Z(I)* reached a maximum, respectively.

The advantage of the standardized Moran’s I is that it can compare the significant spatial patterns of different variables or of the same variable with different calculating parameters. At the global level, Table 1 indicates Hg did not pass the significance test for the global standardized Moran’s I (1.96), and Zn was just at the significance level. Table 2 shows the four metals all pass the significance test, and Cr had the largest spatial dependence, followed by Ni, Hg. Zn had the weakest spatial structure among the four metals.

Compared with the local Moran’s I statistical analyses in Table 2, there were three major variations in Table 1. The first involved spatial clusters and High-low spatial outlier. Their percent considerably decreased for the four heavy metals, particularly Cr and Hg, whereas the absolute value of both Moran’s I and its standardized value for the three types increased distinctly for the four heavy metals. The second related to the spatial randomness (No significant spatial pattern), which increased considerably in percentage for the four heavy metals. These changes implied that spatial clusters and High-low spatial outlier became stronger and more significant; thus, the cores of these local spatial types were highlighted by reducing their structure percent. The third difference was that the Low-high spatial outlier became less significant.

**Table 1:** Spatial autocorrelation characteristics of the four heavy metals at global and local levels based on the distance where the Moran’s I reached maximum.

Heavy metals		Local spatial correlation type					Global
		No significance	High-high	Low-low	Low-high	High-low	
Cr	Moran’s I	0.0872	0.8498	0.7035	−0.1428	−0.2755	0.4801
	Standardized Moran’s I	0.7040	6.7898	5.6228	−1.1319	−2.1906	3.8396
	Percent of spatial types	56.09	14.34	22.2	3.05	4.32	-
Ni	Moran’s I	0.1661	0.9994	0.7527	−0.1724	−0.4386	0.3173
	Standardized Moran’s I	1.1318	6.7756	5.1044	−1.1608	−2.9635	2.1558
	Percent of spatial types	69.94	7.07	12.48	7.96	2.55	-
Zn	Moran’s I	0.1165	0.7315	0.8123	−0.1152	−0.782	0.2924
	Standardized Moran’s I	0.7871	4.9050	5.4464	−0.7650	−5.2300	1.9648
	Percent of spatial types	66.7	8.74	13.46	7.56	3.54	-
Hg	Moran’s I	0.0742	0.9958	0.7823	−0.228	−0.3971	0.2725
	Standardized Moran’s I	0.5223	6.9279	5.4441	−1.5775	−2.7529	1.9009
	Percent of spatial types	67.78	9.63	11.3	8.35	2.95	-

**Table 2:** Spatial autocorrelation characteristics of the four heavy metals at global and local levels based on the distance where the standardized Moran’s I reached maximum.

Heavy metals		Local spatial correlation type					Global
		No significance	High-high	Low-low	Low-high	High-low	
Cr	Moran’s I	0.0296	0.3906	0.3731	−0.2065	−0.1656	0.3333
	Standardized Moran’s I	0.3951	5.0561	4.8293	−2.6525	−2.1245	4.3158
	Percent of spatial types	28.09	19.45	32.81	6.19	13.46	-
Ni	Moran’s I	0.0351	0.4643	0.4287	−0.1674	−0.2148	0.2444
	Standardized Moran’s I	0.3685	4.7486	4.3857	−1.6979	−2.1822	2.5046
	Percent of spatial types	55.30	14.83	20.73	5.01	4.13	-
Zn	Moran’s I	0.0859	0.4629	0.5584	−0.2667	−0.4308	0.2367
	Standardized Moran’s I	0.7422	3.9634	4.7794	−2.2698	−3.6722	2.0308
	Percent of spatial types	56.58	13.16	18.27	5.80	6.19	-
Hg	Moran’s I	0.0064	0.5812	0.3923	−0.2215	−0.2433	0.2054
	Standardized Moran’s I	0.0780	6.1029	4.1226	−2.3114	−2.5401	2.1637
	Percent of spatial types	49.90	16.01	20.53	5.30	8.25	-

The disagreements in the spatial autocorrelation characteristics in Table 1 and Table 2 suggested that the maximum of a raw spatial correlogram can provide a suitable distance for detecting the local highlights of local spatial pattern, and it also agreed with the law that the closer the distance the more similarity. In contrast, the maximum of a standardized spatial correlogram focused on the major structure, with a smoothing effect on the details.

For the four heavy metals, their standardized spatial correlograms had more than one distinct waveform (Figure 3). Figure 3 provides the standardized Moran’s I values of Cr, which were positive at a distance from 1 km to 57 km, 79 km to 140 km. This indicated spatial clusters of similar Cr concentrations at these distance ranges. The standardized Moran’s I values of Cr were negative at a distance from 60 km to 72 km, which implied clusters of dissimilar Cr concentrations; that is, spatial outliers. Similarly, Zn and Hg all showed spatial clusters and spatial outliers over an entire region. 

For the four metals, the amplitudes of spatial clusters were larger than for spatial outliers, indicating that positive spatial autocorrelation dominated at the global level. The same characteristics of the raw and standardized spatial correlograms were the distances where the 0 value first appeared, which were 57 km, 75 km, 57 km, and 55 km for Cr, Ni, Zn, and Hg, respectively (Figure 2, Figure 3). Thus, these were the maximal spatial positive correlation ranges of the corresponding heavy metals.

### 3.2. Spatial Pattern Analysis of Heavy Metals with Geostatistics

Table 3 lists the attributes of the semivariograms for the four heavy metals, their spatial correlation distances were identified as 16.5 km, 20 km, 20 km, and 55 km through trial and error, respectively. The semivariograms of Cr, Ni, Zn, and Hg were well fitted with the exponential model, as indicated by regression coefficients (R^2^) greater than 0.9, which indicated that Cr, Ni, Zn, and Hg had stronger spatial structure. The nugget/sill ratio of Cr, Ni, Zn, and Hg ranged from 34.2% to 48.9%, suggesting moderate spatial dependence, which indicates that the spatial variability of Cr, Ni, Zn, and Hg may be affected by intrinsic (soil formation factors, such as soil parent materials) and extrinsic factors (soil management practices, such as fertilization and pesticides). The spatial dependence of Cr is the strongest, followed by Ni, Zn, and then Hg. There was agreement in the spatial dependence of Cr and Ni metals between the geostatistics analysis and the spatial autocorrelation analysis, but disagreement for Zn and Hg. Comparing the significant spatial pattern of different variables, spatial autocorrelation analysis is preferred over geostatistics in spatial dependence because semivariance cannot test the significance of spatial dependence.

The ranges (spatial correlation distances) of Cr, Zn, and Hg are around 60 km, which indicated the spatial distribution of these three heavy metals may be similar, and the source may be the same. The spatial variability of Hg was significant, which indicated that the concentrations of Hg in soils were mainly affected by random factors (human activities).

The spatial correlation distances of Cr, Ni, Zn, and Hg from geostatistics were 59.55 km, 94.50 km, 65.79 km and 65.10 km, respectively (Table 3). These were larger than the corresponding ranges obtained from spatial autocorrelation analysis. The difference arose in part from the fact that geostatistics includes a mixture of positive and negative correlation [17].

**Table 3:** Semivariogram models for Cr, Ni, Zn, and Hg and their parameters (range, km).

Heavy metals	Model	Nugget (C_0_)	Sill (C_0_ + C)	Range (A_0_)	Nugget/sill (C_0_/(C_0_ + C))/%	R^2^	RSS
Cr	Exponential	0.0251	0.0733	59.55	34.2	0.980	1.21 × 10^−5^
Ni	Exponential	0.0596	0.1423	94.50	41.9	0.972	3.52 × 10^−5^
Zn	Exponential	0.0377	0.0801	65.79	47.1	0.930	3.80 × 10^−5^
Hg	Exponential	0.5010	1.0250	65.10	48.9	0.969	5.20 × 10^−3^

### 3.3. The Comparative Analysis of Estimation Accuracy for Global Heavy Metals

According to the spatial correlograms, four representative distances were selected: 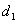 and 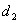 represented the distance where the Moran’s I and the standardized Moran’s I, 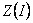 reached maximum, respectively. 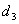 was the maximal positive correlation distance for Cr, Ni, Zn, and Hg, and 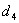 was the sum distance of the positive and negative correlation. Table 4 lists these characteristic distances. There was no 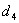 for Ni because no distinct negative correlation range was found between the two positive correlation ranges. 

**Table 4:** The four characteristic distances of Cr, Ni, Zn, and Hg (km).

Heavy metals	Distance 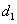	Distance 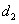	Distance 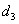	Distance 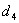
Cr	6	16	57	76
Ni	4	10	75	-
Zn	4	7	57	78
Hg	4	11	55	91

A variogram plot can be acquired by calculating variograms at different lags. For irregular sampling, the active lag distance is often represented by a distance band. Generally, the distance band was adjusted repeatedly for higher match between the theoretical model and the experimental semivariance. In this study, in order to effectively and quickly find the suitable active lag distance, we tried to use the distances parameters extracted from the Moran’s I analysis as an auxiliary tool. Therefore, the four characteristic distances were tested as the active lag distance to fit the semivariance and produce spatial interpolation, and these were labeled by model 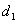, 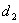, 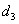, 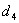, respectively. Because the Moran’s I analysis based on 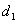 and 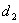 can highlight the local (often the spatial outliers) and major spatial structure and the complexity of spatial variables, the nested model of 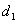 and 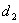 was used to describe the spatial variability of heavy metals at local and global scales. It is worth noting that model 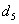 was the traditional geostatistics model in the following.

**Figure 4:**
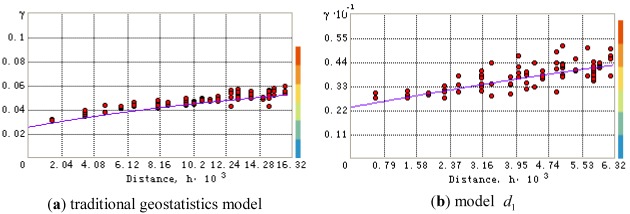
The scatter plots and fitted model based on the traditional geoststistics model (**a**) and model 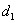 (**b**) (distance, m).

Take Cr as a case. Semivariogram *γ(h)* for Cr was calculated and the scatter plot of *γ(h)* *vs.* 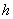 was generated. The different theoretical semivariance models were used to try with the best fitting value. The smallest nugget values of mean prediction error (ME) close to 0 and root-mean-square standardized prediction errors (RMSSE) close to 1 were selected. Figure 4 presents the scatter plots and fitted model based on the traditional geoststistics model and model 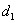. According to the scatter plots, the values ME and RMSSE were calculated. The ME values of traditional geostatistics model and model 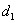 were 0.234, 0.1015, and the RMSSE were 1.119 and 1.075, respectively. Therefore, the theoretical semivariance of model 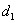 was better than traditional geostatistics model. For Ni, Zn, and Hg, The selection of the semivariance were similar to Cr.

The spatial interpolation maps of the four heavy metals were conducted using the ordinary kriging method based on model 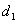, 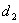, 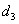, 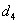, 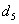, and the nested model of 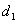 and 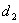. The cross-validation method was used to validate the parameters of these models. Evaluation indices from cross-validation of spatial maps of the four heavy metals are given in Table 5. For Cr, Ni, Zn, and Hg, except for the nested model of 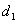 and 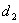, the MAE, RMSE, and MAE values of model 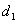 and model 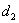 were the smallest, which indicated that semivariance parameters obtained from fitting of experimental semivariogram values were reasonable and spatial prediction using semivariogram parameters is better than other models. The values of model 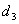 and 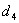 were close and the largest, implying unsuitable semivariogram parameters. The values of model 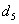 were close to the values of model 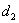, which were the third smallest. This suggested that the dominant spatial pattern can be captured through traditional geostatistics. Compared with model 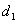, the values of the MAE, RMSE, and MAE of the nested model of 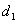 and 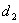 were smaller, which indicated that the nested model can further improve the global prediction. It may be caused by the model 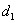 being able to highlight local spatial patterns while the model 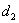 focuses on the global dominant spatial pattern. Thus, their coupling may allow fusion of the details and the dominating spatial patterns of heavy metals. Table 5 shown that the best model was the nested model of 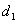 and 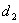, followed by model 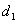, 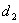, 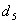. Therefore, the Moran’s I analysis can help to provide the better semivariogram parameters and improve estimation accuracy.

Table 6 lists the statistics results of measured and predicted heavy metals concentrations. After spatial interpolation, the mean concentrations of Cr, Ni, Zn, and Hg for different models were similar to those of the measured values. The minimum values were larger than those of the measured value, while the maximum values, standard deviations, and CVs were smaller than those of the measured value. The result indicated that all four interpolation methods caused a degree of compression due to the smoothing effect of kringing interpolation. When the model 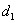 and the nested model 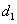 plus 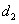 were used, the data ranges were wider, and the standard deviations and CVs were larger than when other models were used. This indicated that the model 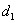 and the nested model 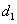 plus 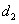 can reduce the smoothing effect of kriging and improve the estimatiom accuracy, especially the nested model of 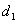 and 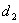.

**Table 5:** Evaluation indices of the interpolation maps of heavy metals.

	Evaluation indices	Cr	Ni	Zn	Hg
Model 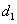	MAE	6.37	1.45	8.66	0.0935
	RMSE	10.56	3.20	12.56	0.2170
	MSE	111.57	10.27	157.66	0.0471
Model 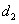	MAE	6.47	3.55	9.92	0.1195
	RMSE	10.81	7.29	14.34	0.2683
	MSE	116.76	53.19	205.53	0.0720
Model 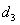	MAE	7.75	5.44	12.16	0.1147
	RMSE	12.73	9.91	17.42	0.2540
	MSE	161.97	98.20	303.61	0.0645
Model 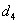	MAE	7.66	-	12.53	0.1351
	RMSE	12.59	-	17.88	0.2817
	MSE	158.51	-	319.55	0.0794
Model 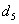	MAE	7.06	4.83	10.86	0.1160
	RMSE	11.69	9.16	15.66	0.2557
	MSE	136.73	83.86	245.16	0.0654
Model 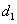 plus 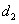	MAE	5.31	1.17	7.67	0.0918
	RMSE	8.90	2.59	11.09	0.2291
	MSE	79.19	6.73	123.03	0.0525

**Table 6:** Statistics results of measured and predicted heavy metals concentrations (mg·kg^−1^).

Heavy metals		Mean	Minimum	Maximum	Range	Standard Ddeviation	CV (%)
Cr	Measured value	60.75	31.60	300.00	268.40	20.49	33.73
	Model 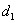	60.57	39.45	156.59	117.14	14.28	23.57
	Model 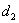	60.56	39.93	142.62	102.69	13.90	22.95
	Model 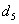	60.50	40.69	136.41	95.72	13.50	22.32
	Model 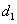 plus 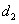	60.82	38.19	162.12	123.94	15.18	24.95
Ni	Measured value	28.49	8.87	203.38	194.51	11.25	39.49
	Model 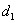	28.42	10.16	139.08	128.91	8.64	30.42
	Model 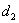	28.39	13.45	59.34	45.89	5.88	20.72
	Model 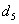	28.44	15.04	44.72	29.68	4.86	17.10
	Model 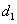 plus 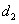	28.55	10.10	147.14	137.04	9.15	32.07
Zn	Measured value	76.27	28.50	221.62	193.12	21.03	27.57
	Model 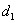	76.26	45.44	144.82	99.38	12.31	16.14
	Model 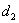	76.20	47.76	128.93	81.17	11.18	14.67
	Model 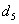	76.14	49.99	117.25	67.27	10.37	13.62
	Model 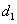 plus 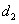	76.55	43.75	159.64	115.88	13.43	17.55
Hg	Measured value	0.2175	0.0005	4.2900	4.2895	0.3210	147.59
	Model 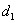	0.2113	0.0219	1.6256	1.6037	0.1602	75.80
	Model 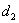	0.2035	0.0509	0.8837	0.8328	0.1177	57.87
	Model 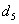	0.2072	0.0485	1.1382	1.0897	0.1327	64.04
	Model 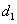 plus 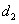	0.2129	0.0314	1.1547	1.1233	0.1510	70.90

The above results shown that the characteristic distances provided by Moran’s I analysis are feasible for improving the spatial estimation accuracy, but the mathematical proof of the methodology was not explored here. In addition, ordinary kriging, the most basic and commom spatial interpolation method, was used to, other kriging model such as indicator kriging (which makes no assumption of normality) with Moran’s I analysis can be further examined for the possible.

### 3.4. The Impact on Pollution Status of Heavy Metals

In order to understand the impact of the spatial interpolation on the pollution status of heavy metals, a single-factor method was used to assess the pollution status, based on the critical value of “Chinese Environmental Quality Standard for Soils” (GB 15618-1995). The pollution status was classified, according to a single-factor pollution index, as unpolluted or polluted [26]. After spatial interpolation using model 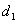, 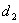, the nested model of 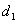 and 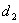, and the traditional geostatistics model 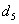, the pollution status of both measured and predicted values of the samples were assessed. In Table 7, the rows are the pollution status based on predicted values, while the columns are the pollution status based on measured values. Thus, in each block composed by heavy metal and prediction model, data in the left diagonal present the agreement in pollution status between the measured and predicted values, while those in the right diagonal present the disagreement in pollution status between the measured and predicted values.

**Table 7:** The sample agreements in pollution status between ground measure and interpolation (%).

		Cr		Ni		Zn		Hg	
		Polluted	Unpolluted	Polluted	Unpolluted	Polluted	Unpolluted	Polluted	Unpolluted
Model *d*_1_	Polluted	0.10		2.36				3.05	0.49
	Unpolluted	0.59	99.31	1.57	96.07	0.10	99.90	3.24	93.22
Model *d*_2_	Polluted			1.28	0.29			1.87	0.39
	Unpolluted	0.69	99.31	2.65	95.78	0.10	99.90	4.42	93.32
Model *d*_5_	Polluted			0.49	0.29			1.96	0.39
	Unpolluted	0.69	99.31	3.44	95.78	0.10	99.90	4.32	93.32
Model *d*_1_ plus *d*_2_	Polluted	0.10		3.05				3.24	0.59
	Unpolluted	0.59	99.31	0.88	96.07	0.10	99.90	3.05	93.12

For Zn, 0.1% of measured samples were in pollution status, and these became unpolluted after spatial interpolation using all models, although the nested model of 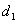 and 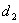 improved the Zn global prediction accuracy (Table 7). For Cr, 0.69% of measured values were in pollution status, and these samples were underestimated and became unpolluted after interpolation using models 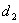 and traditional geostatistics model 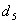. However, after interpolation using model 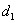 and the nested model of 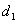 and 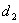, there were still 0.10% of samples remained pollution status. Before and after interpolation, the changes of samples pollution status were more complicated for Ni and Hg, as there was not only an underestimation effect, but also an overestimation effect. These two effects were severe after interpolation using models 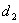 and 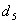 (Table 7). For Ni, 3.93% of measured samples were in pollution status. After spatial interpolation, the model 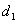 avoided the overestimation effect and reduced the underestimation effect from 3.44% to 1.57% compared with the model 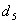. The nested model of 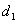 and 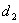 showed that there were still 3.05% of samples remained pollution status, but only 0.88% of samples were changed from polluted to unpolluted status after interpolation.

For Hg, 6.29% of measured samples were in pollution status. Compared with the model 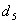, the percent of samples remained pollution status increased from 1.96% to 3.05% after interpolation using the model 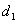, and the percent of samples changed from polluted to unpolluted reduced from 4.32% to 3.24%. Thus, it avoided designating 1.08% of polluted samples wrongly as unpolluted. Models 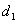 and 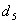 also had the overestimation effect, the percent of samples changed unpolluted to polluted were 0.39% and 0.49%, respectively, therefore, 0.10% of unpolluted samples were wrongly identified as polluted. Compared with model 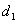, the percent of samples remained pollution status increased by 0.19%, and the percent of samples changed from unpolluted to polluted increased by 0.10% after interpolaton using the nested model of 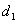 and 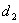.

The results of pollution status assessment of samples showed that, compared with the traditional geostatistics model 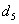, the method combining geostatistics with Moran’s I analysis (models 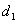, 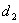) can avoid effectively the underestimation and overestimation effect so that more samples remain original pollution status. 

In general, heavy metal polluted soil samples, as a small probability event, would be underestimated by interpolation, which is exemplified by Zn and Cr in this study. If the distance where the Moran’s I reached maximum (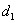) was used to optimize the semivariance, this underestimation effect can be slightly reduced. With the increase of the polluted samples and a significant local spatial pattern appeared, the effect of reducing underestimation was more significant, such as Ni and Hg in this study. If the distances where the Moran’s I and the standardized Moran’s I, 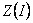 reached maximum (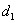 and 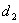) were combined to optimize the semivariance, the underestimation effect can be reduced further. The reason for this may be that the optimality of semivariance using 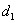 can highlight the most significant local spatial patterns and reflects the more abnormal information of the polluted samples. Thus, the nested model would better balance the details and dominating spatial patterns. But, there also was an overestimation effect. Because most of the overestimation errors happened near actual polluted samples, making the potentially high-risk areas, in terms of soil heavy metal pollution, a little overestimation may be better than more underestimation. By comparing all the models 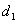, 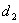, 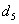, the nested model of 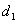 and 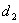, the nested model was defined as the optimality model.

As mentioned previously, spatial autocorrelation analysis was adopted for the optimality of semivariance by simply deleting the spatial outliers. However, in pollution studies, this may cause severe hypercorrectness when the spatial outliers are in fact reasonable. If global dominating spatial patterns are the focus, then standardized spatial correlogram can provide the spatial correlation distance for the optimality of semivariance. In contrast, if abnormal situations or details are the key, such as evaluation of the soil heavy metal pollution, then raw spatial correlogram can provide the information to help the optimality of semivariance. Moreover, a nested model that fuses both the details and the dominating spatial patterns can provide an even better prediction.

In the current study, for Hg metal element, there still was a large gap of assessment accuracy between the nested model interpolation and the measured values. A greater improvement in assessment accuracy may occur if zonal geostatistics are interpolated according the spatial distribution of the local Moran’s I spatial pattern types. In addition, as this study primarily focused on the spatial autocorrelation analysis for the optimality of semivariance, the ordinary kriging type may not have been the optimal for the estimation accuracy. 

### 3.5. The Spatial Distribution of Heavy Metals Using the Nested Model Interpolation

**Figure 5:**
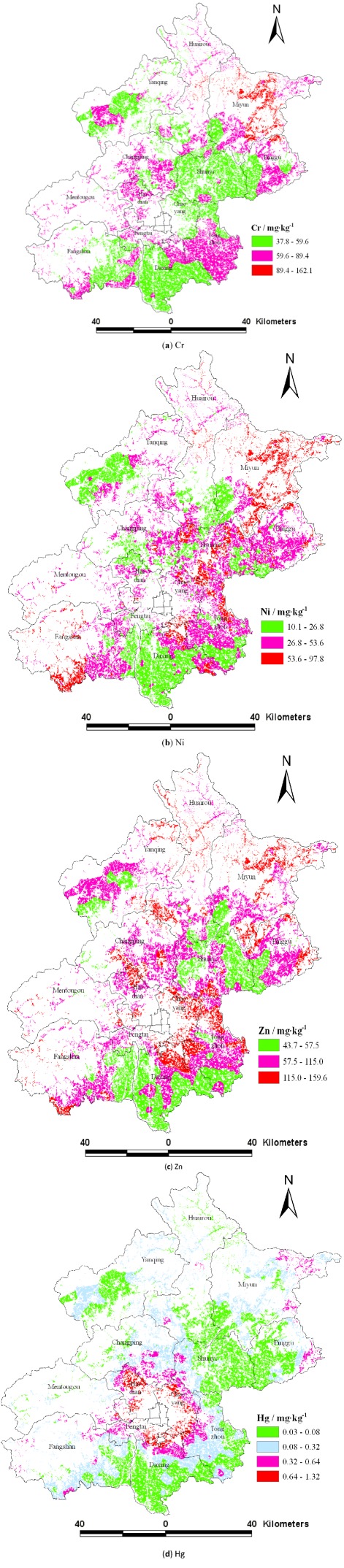
Distribution maps of heavy metals based on the nested model of 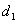 and 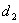 (**a**) Cr, (**b**) Ni, (**c**) Zn, (**d**) Hg.

Figure 5 shows the spatial distribution of heavy metal concentrations interpolated using the optimality model, that is the nested model 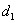 and 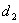. Heavy metals concentrations are separated into classes according to the background values of soil heavy metals of Beijing and their multiples to highlight the spatial differences of different classes.

Cr concentrations in the study areas were greater than the background value (29.8 mg·kg^−1^), and areas where Cr concentrations were three times the background value were observed in northeast Beijing (Figure 5(a)). The areas where Ni concentrations were lower than the background value (26.8 mg·kg^−1^) were more, and areas where Ni concentrations were twice the background value were found in the Miyun, Shunyi, and Daxing districts (Figure 5(b)). Areas where Zn concentrations were lower than the background value (57.5 mg·kg^−1^) were in the Daxing and Fangshan districts, and the areas that Zn concentrations were twice of the background value were in the Daxing, Huairou, Pinggu, and Fangshan districts (Figure 5(c)). The areas that Hg concentrations were lower than background value (0.08 mg·kg^−1^) were in Daxing and Shunyi districts, and the areas that Hg concentrations exceeded 0.32 mg·kg^−1 ^(four times of Hg background value) were primarily distributed in the urban fringe around the city, while a few areas with higher levels were also observed in the Miyun, Pinggu and Fangshan districts (Figure 5(d)). The background value was the original content of soil heavy metals which was not or rarely influenced by human activities. However, the spatial distribution of the four heavy metals showed that human activities had caused the content of heavy metals to be more than the background value and to accumulate. Three major areas were identified that should be received more attention. One is the northeast region of Beijing, where mining operations are responsible for the enrichment in heavy metals. The second is the southeast part of Beijing where wastewater irrigation has strongly changed the content of metals in soils, particularly those of Cr and Zn. The last is the urban fringe around the city, where Hg has had a significant increase.

## 4. Conclusions

Both geostatistics and spatial autocorrelation analysis can evaluate the spatial patterns of heavy metals. However, the two methods have their advantages and disadvantages. Geostatistics can provide a technique of semivariance to quantify the spatial patterns of soil parameters, but the fitting of variogram is influenced by subjective factors, and it will affect the kriging estimation. On the other hand, the Moran’s I analysis just can provide some spatial autocorrelation distances of variable, which have the same meaning as the range calculation from the variogram, so in this paper we tried to use this information to help calculate the semivariance in geostatistics and produce spatial interpolation to improve the accuracy of traditional geostatistics. This is the method combining geostatistics with Moran’s I analysis.

According to spatial correlogram of Moran’s I analysis, four characteristics distances were obtained and used as the active lag distance to calculate the semivariance. The resulted showed that the fitting accuracy of semivariance based on the distances where the Moran’s I and the standardized Moran’s I, *Z(I)* reached maximum were better than traditional geostatistics, because the Moran’s I analysis based on the two distance can detect the local spatial and major spatial pattern of heavy metals, respectively. Then, the spatial interpolation was produced based on the two distances. By comparing the values MAE, RMSE, and MSE, and the pollution status of measured and predicted values, the results showed that the estimation accuracy of the method combining geostatistics with Moran’s I analysis were better than the traditional geostatistics. In addition, because of the complexity of spatial data, the nested model, which is coupled by the distances where the Moran’s I and the standardized Moran’s I, *Z(I)* reached a maximum, was used to calculate the semivariance and produce spatial interpolation, the results showed that the nested model was the optimality and improve the fitting and estimation accuracy further. Consequently, the Moran’s I analysis can be used as a useful complement to geostatistics and produce a high quality spatial interpolation maps of heavy metals. Based on the interpolation maps produced using the nested model, areas of high concentrations of heavy metals can be identified, this is very important for risk assessment of environmental and pollution remediation.
